# Restoration of Contralateral Representation in the Mouse Somatosensory Cortex after Crossing Nerve Transfer

**DOI:** 10.1371/journal.pone.0035676

**Published:** 2012-04-20

**Authors:** Haruyoshi Yamashita, Shanlin Chen, Seiji Komagata, Ryuichi Hishida, Takuji Iwasato, Shigeyoshi Itohara, Takeshi Yagi, Naoto Endo, Minoru Shibata, Katsuei Shibuki

**Affiliations:** 1 Department of Neurophysiology, Brain Research Institute, Niigata University, Niigata, Japan; 2 Department of Plastic Surgery, Faculty of Medicine, Niigata University, Niigata, Japan; 3 Department of Orthopedic Surgery, Faculty of Medicine, Niigata University, Niigata, Japan; 4 Beijing Jishuitan Hospital, Beijing, People's Republic of China; 5 Division of Neurogenetics, National Institute of Genetics, Mishima, Japan; 6 Laboratory for Behavioral Genetics, RIKEN Brain Science Institute, Wako, Japan; 7 KOKORO-Biology Group, Laboratories for Integrated Biology, Graduate School of Frontier Biosciences, Osaka University, Osaka, Japan; Imperial College London, United Kingdom

## Abstract

Avulsion of spinal nerve roots in the brachial plexus (BP) can be repaired by crossing nerve transfer via a nerve graft to connect injured nerve ends to the BP contralateral to the lesioned side. Sensory recovery in these patients suggests that the contralateral primary somatosensory cortex (S1) is activated by afferent inputs that bypassed to the contralateral BP. To confirm this hypothesis, the present study visualized cortical activity after crossing nerve transfer in mice through the use of transcranial flavoprotein fluorescence imaging. In naïve mice, vibratory stimuli applied to the forepaw elicited localized fluorescence responses in the S1 contralateral to the stimulated side, with almost no activity in the ipsilateral S1. Four weeks after crossing nerve transfer, forepaw stimulation in the injured and repaired side resulted in cortical responses only in the S1 ipsilateral to the stimulated side. At eight weeks after crossing nerve transfer, forepaw stimulation resulted in S1 cortical responses of both hemispheres. These cortical responses were abolished by cutting the nerve graft used for repair. Exposure of the ipsilateral S1 to blue laser light suppressed cortical responses in the ipsilateral S1, as well as in the contralateral S1, suggesting that ipsilateral responses propagated to the contralateral S1 via cortico-cortical pathways. Direct high-frequency stimulation of the ipsilateral S1 in combination with forepaw stimulation acutely induced S1 bilateral cortical representation of the forepaw area in naïve mice. Cortical responses in the contralateral S1 after crossing nerve transfer were reduced in cortex-restricted heterotypic GluN1 (NMDAR1) knockout mice. Functional bilateral cortical representation was not clearly observed in genetically manipulated mice with impaired cortico-cortical pathways between S1 of both hemispheres. Taken together, these findings strongly suggest that activity-dependent potentiation of cortico-cortical pathways has a critical role for sensory recovery in patients after crossing nerve transfer.

## Introduction

Amelioration of functional disabilities after peripheral nerve injury remains extremely challenging [1]. Accidental mechanical forces applied to the arm result in avulsion of proximal spinal nerve roots from the spinal cord. Avulsion injuries to the brachial plexus (BP) can be repaired by nerve transfer between injured nerve ends and the accessory nerve or intercostal nerve ipsilateral to the lesioned side using a nerve graft, although the functional recovery after the operation is not necessarily satisfactory [2]. Crossing nerve transfer, which connects injured nerve ends to the healthy BP contralateral to the lesioned side, has also been used as an alternative approach to repair avulsion injuries to BP [3], [4]. This surgery has been shown to result in functional reorganization of the motor cortex in both hemispheres [5], [6]. Furthermore, sensory recovery of an injured/repaired hand suggests that functional reorganization is produced in the primary somatosensory cortex (S1) of both hemispheres after crossing nerve transfer. S1 reorganization within a hemisphere is induced by sensory loss [7], [8], and direct cortical stimulation produces potentiation of cortico-cortical pathways connecting S1 in both hemispheres [9]. Therefore, it is likely that activity-dependent neural plasticity, which involves S1 in both hemispheres, could be induced after crossing nerve transfer. The present study analyzed this possibility in an experimental mouse model.

Genetically manipulated strains of mice are helpful for investigating molecular mechanisms that underlie cortical changes after crossing nerve transfer. Neural plasticity is expected to played a critical role in functional recovery from BP injury [1], [2], and many types of activity-dependent plasticity are dependent on NMDA receptors [10], [11], [12]. Therefore, the present study investigated cortical responses after crossing nerve transfer in cortex-restricted heterozygous of GluN1 (NMDAR1) subunit knockout mice [13]. In these mice, approximately 50% of the functional NMDA receptors are specifically lost in cortical excitatory neurons, although no apparent abnormalities have been observed during development [13]. Through the use of this knockout mouse model, it is possible to analyze whether NMDA receptor-dependent synaptic potentiation in cortical synapses plays an essential role in functional recovery after crossing nerve transfer.

Another merit in the use of mice is that cortical activity can be investigated using transcranial imaging, with no surgical damage to the cortex [14]. Activity-dependent fluorescence signals, which are derived from mitochondrial flavoproteins [15], [16] are useful for transcranial imaging [17], [18]. In rats, stimulation applied to the forepaw results in cortical activities in both hemispheres [9], while the S1 ipsilateral to the stimulated side is only weakly activated in mice [19]. Stimulation to the forepaw, which connects to the brain via the crossing nerve graft only, is expected to primarily activate the ipsilateral S1. Therefore, the appearance of cortical activity in the contralateral S1 is an indicator for S1 functional reorganization in both hemispheres. Therefore, the present study analyzed cortical changes induced by crossing nerve transfer as well as the underlying mechanisms.

## Results

### Bilateral cortical representation after crossing nerve transfer

**Figure 1:**
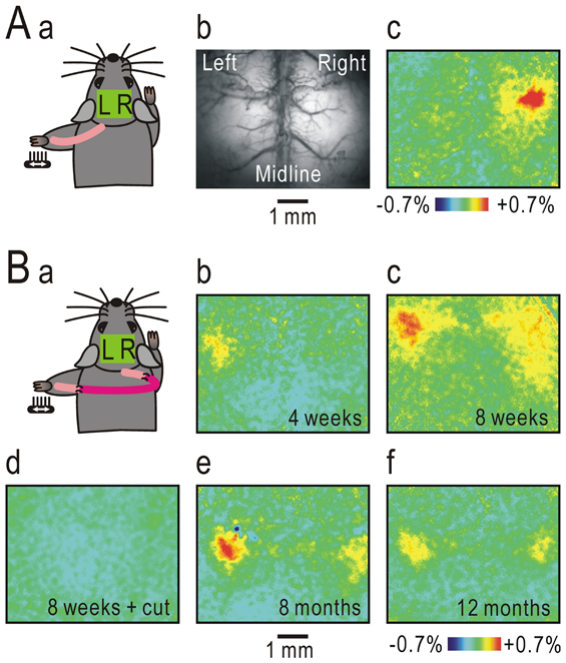
Cortical responses before and after crossing nerve transfer. (A) Cortical responses elicited by vibratory stimulation applied to the left forepaw in a naïve mouse. In the left diagram (a), peripheral nerves are shown on the back of a mouse to avoid left-right confusion. The middle panel (b) shows the original fluorescence image. In the right panel (c), neural activity is apparent in the contralateral right S1, while the ipsilateral left S1 is only weakly activated. (B) Cortical responses after crossing nerve transfer. The diagram (a) and cortical responses elicited by vibratory stimulation applied to the left forepaw at 4 weeks (b), 8 weeks (c and d), 8 months (e) and 12 months (f) after crossing nerve transfer. The cortical responses shown in (c) were almost completely lost after the nerve graft was cut in the same mouse (d).

**Figure 2:**
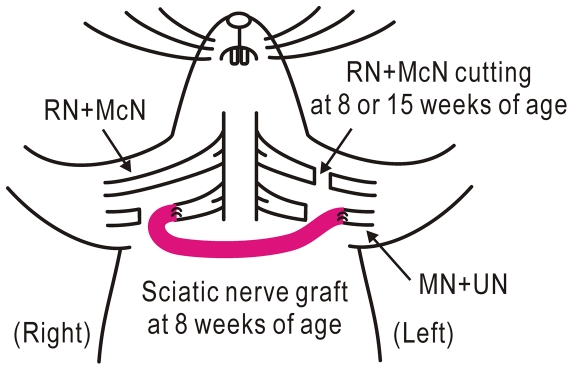
Crossing nerve transfer surgery. The cut ends of the left median (MN) and ulnar nerves (UN) were connected to the right brachial plexus (BP) via a sciatic nerve graft at 8 weeks of age. The left radial (RN) and musculocutaneous nerves (McN) were cut at the same time, or at 15 weeks of age (sequential nerve cut). Imaging was performed at 16 weeks of age.

**Figure 3:**
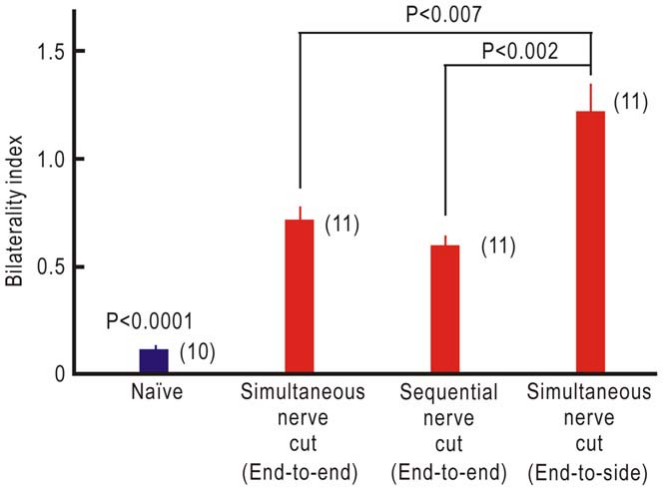
Bilaterality index in mice with or without crossing nerve transfer. The bilaterality index in mice with crossing nerve transfer surgery using three different methods is significantly greater than that in naïve mice (P<0.0001, respectively). The bilaterality index is not significantly different between mice operated in an end-to-end fashion, with or without sequential nerve cut. However, the index in mice operated in an end-to-side fashion was significantly larger than that in mice operated in an end-to-end fashion with (P<0.002) or without sequential nerve cut (P<0.007). Numbers in the parentheses show the numbers of mice.

In naïve mice, vibratory stimulation applied to the palm of the forepaw resulted in localized cortical responses in the S1 contralateral to the stimulated side, while the ipsilateral S1 was only weakly activated (Fig. 1A). The sensory information from the forepaw palm is mainly mediated via the median and ulnar nerves. However, the radial and musculocutaneous nerves are also involved [19]. In crossing nerve transfer, therefore, the peripheral cut ends of the left median and ulnar nerves were connected to the central cut ends of the contralateral BP using a sciatic nerve graft in an end-to-end fashion, and the remaining radial and musculocutaneous nerves were cut (simultaneous nerve cut, Fig. 2). At four weeks after this operation, responses began to appear in the ipsilateral S1, but almost no response was found in the contralateral S1 (Fig. 1Bb). At eight weeks after the operation, left forepaw stimulation resulted in a clear cortical response in the ipsilateral S1, and cortical activity was also found in the contralateral S1 (Fig. 1Bc). These cortical responses were completely abolished by cutting the nerve graft (Fig. 1Bd). Bilateral cortical representation was also found at 8 and 12 months after the operation (Fig. 1Be and Bf), although skull transparency was reduced in these older mice. To determine the extent of bilateral cortical representation in S1, the bilaterality index was defined as the ipsilateral response amplitude normalized by contralateral value in naïve mice, and as the contralateral response amplitude normalized by ipsilateral value in mice with crossing nerve transfer. The bilaterality index at 8 weeks after crossing nerve transfer was significantly larger than in naïve mice (P<0.0001, Fig. 3), indicating that bilateral cortical representation in the S1 forepaw area was clearly established at 8 weeks after crossing nerve transfer.

Since sensory information from the injured forepaw area is lost after crossing nerve transfer, the contralateral S1 forepaw area could be occupied by afferent inputs originating outside the forepaw area [7], [8]. To estimate this effect on the cortical changes after crossing nerve transfer, the left radial and musculocutaneous nerves were maintained up to 7 week after crossing nerve transfer of the median and ulnar nerves, and cut at 1 week before the imaging experiments. However, mice operated with this sequential nerve cut exhibited similar bilateral somatosensory responses, and the bilaterality index was not significantly different from mice with simultaneously resected left radial and musculocutaneous nerves and crossing nerve transfer (Fig. 3).

One of the drawbacks to crossing nerve transfer is that the healthy BP must be cut during surgery. To induce as little damage as possible to the healthy BP, the crossing nerve transfer was also performed in an end-to-side fashion: the nerve graft was sutured to a window of the epineural sheath on the right healthy BP [20]. Bilateral cortical representation of the left forepaw was clearly observed at 8 weeks after this operation, and the bilaterality index was significantly larger than in mice with end-to-end surgery, with or without sequential cut of the left radial and musculocutaneous nerves (P<0.002 and 0.007, respectively; Fig. 3). These findings suggest that crossing nerve transfer in an end-to-side fashion restored neural activity in the contralateral S1 more efficiently than in an end-to-end fashion. In the following experiments, however, crossing nerve transfer in an end-to-end fashion was utilized, because this surgery is easier to perform in mouse models.

### Photo-inactivation of S1 cortical responses

**Figure 4:**
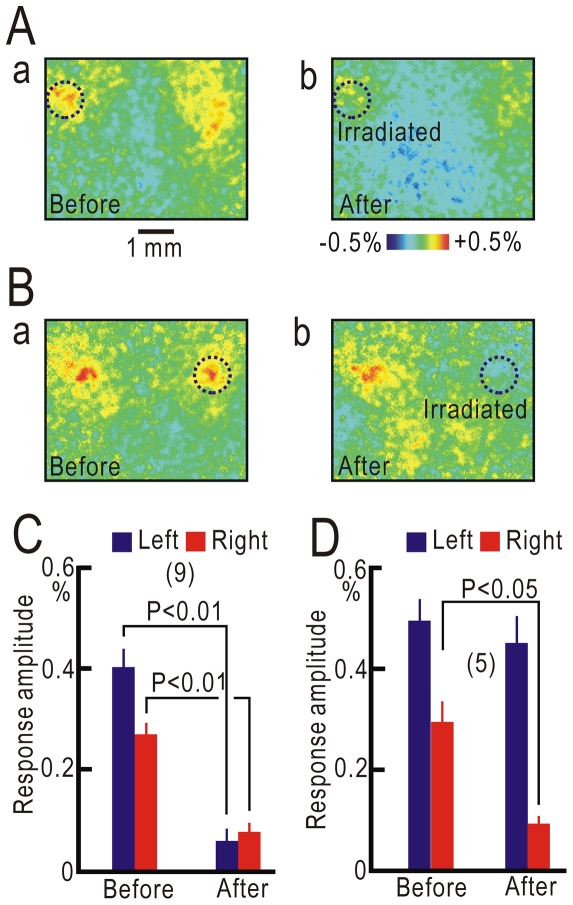
Photo-inactivation of cortical responses after crossing nerve transfer. (A) Cortical responses before (a) and after (b) photo-inactivation of ipsilateral left responses in the same mouse. (B) Cortical responses before (a) and after (b) photo-inactivation of contralateral right responses in the same mouse. (C) Amplitudes of cortical responses before and after photo-inactivation of the ipsilateral S1. (D) Amplitudes of cortical responses before and after photo-inactivation of the contralateral S1.

Cortical responses elicited by left forepaw stimulation were observed in the ipsilateral S1 at 4 weeks and the contralateral S1 at 8 weeks after crossing nerve transfer, respectively (Fig. 1B). This order suggests that the ipsilateral S1 primary responses could be mediated to the contralateral S1 via cortico-cortical pathways. To test this hypothesis, responses in the ipsilateral S1 were suppressed by transcranial photobleaching of flavoproteins, which resulted in photo-inactivation of neural activity [21], because local cortical activity has been shown to be suppressed using this method, with no surgical damage to the cortex. When the activated cortical area in the ipsilateral S1 was irradiated with blue laser light for 90 min, cortical responses in the irradiated area were significantly suppressed (P<0.01, Fig. 4A, C). In addition, cortical responses in the contralateral S1 were also significantly suppressed (P<0.01), although the area was not directly exposed to the laser. When the contralateral S1 response area was initially irradiated, only the contralateral responses were significantly suppressed (P<0.05, Fig. 4B, D). These results suggest that cortical responses in the ipsilateral S1 were conveyed to the contralateral S1 via cortico-cortical pathways.

### Direct cortical stimulation

**Figure 5:**
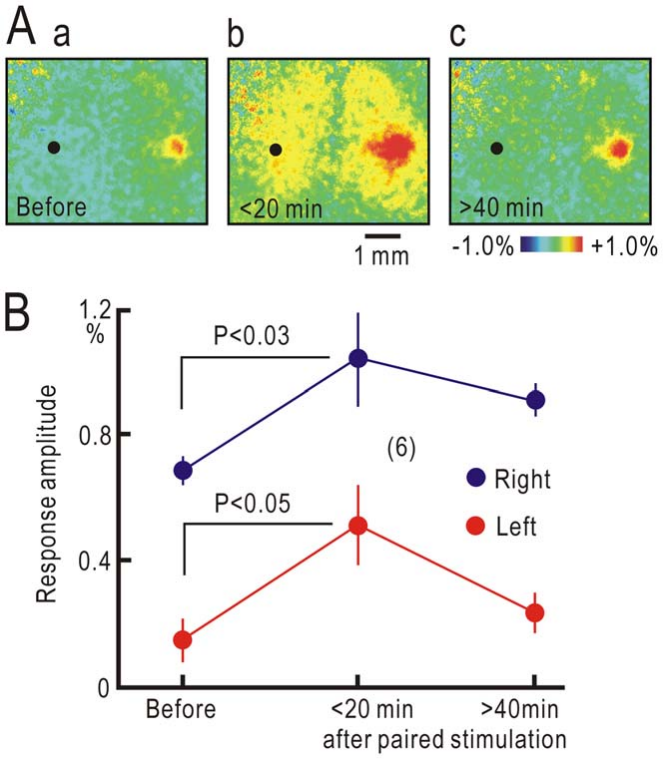
Bilateral cortical representation induced by direct cortical stimulation. (A) Cortical responses elicited by left forepaw stimulation before (a), within 20 min (b) and more than 40 min (c) after cessation of direct stimulation applied to the ipsilateral left S1 paired with left forepaw stimulation in the same mouse. These experiments were performed in naïve mice. The black dot in (a–c) shows the direct cortical stimulation site. (B) Amplitudes of cortical responses elicited by left forepaw stimulation in the ipsilateral left S1 (red) and contralateral right S1 (blue).

In our previous study, cortico-cortical pathways between S1 in both hemispheres are temporarily potentiated by high-frequency cortical stimulation applied to the ipsilateral S1 of anesthetized rats [9]. Therefore, the present study tested whether bilateral cortical representation was acutely reproduced in naïve mice via direct stimulation of the ipsilateral left S1 paired with vibratory stimulation applied to the left forepaw. Cortical responses in the ipsilateral S1 were significantly potentiated within 20 min after cessation of cortical stimulation (P<0.05, Fig. 5A, B). The contralateral S1 responses were also significantly potentiated (P<0.03, Fig. 5A, B). The magnitude of cortical responses typically returned to pre-stimulation levels within 40 min after cessation of cortical stimulation. However, potentiated cortical responses were maintained for more than 80 min in some mice. These results suggest that bilateral cortical representation after crossing nerve transfer could be due to activity-dependent potentiation of cortico-cortical pathways.

### Roles of NMDA receptors in bilateral cortical representation

**Figure 6:**
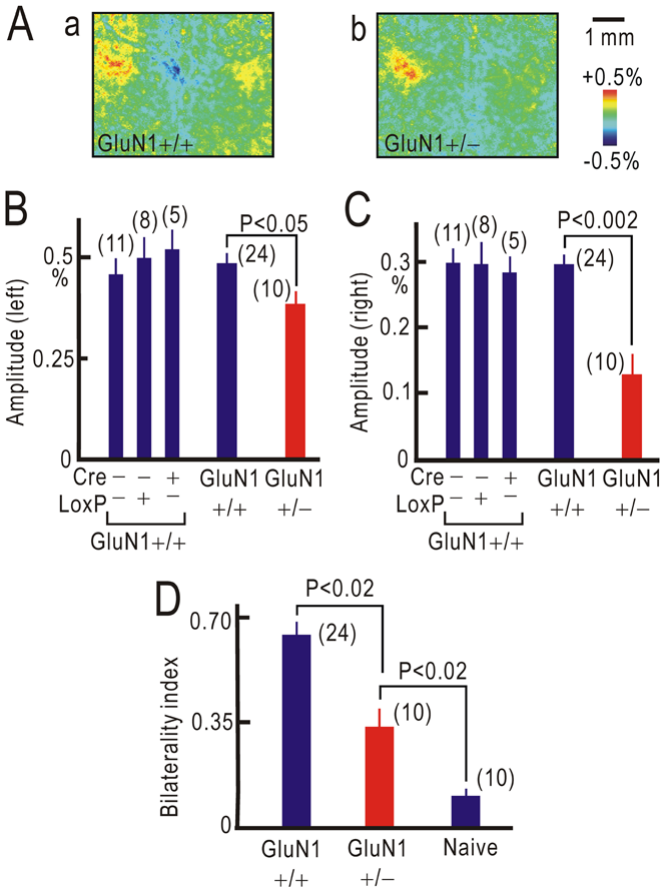
Crossing nerve transfer in cortex-restricted heterozygous GluN1 knockout mice. (A) Cortical responses elicited by left forepaw stimulation after crossing nerve transfer in control GluN1 +/+ (a) and cortex-restricted GluN1 +/− mice (b). (B) Amplitudes of cortical responses recorded in the ipsilateral left S1 after crossing nerve transfer. Control GluN1 +/+ mice expressed Cre alone, LoxP alone, or neither, and cortex-restricted GluN1 +/− mice expressed both Cre and LoxP. (C) Amplitudes of cortical responses recorded in the contralateral right S1 after crossing nerve transfer. (D) Bilaterality index after crossing nerve transfer in control GluN1 +/+ and cortex-restricted GluN1 +/− mice. The index in naïve mice is also shown for comparison.

Since NMDA receptors play important roles in various types of synaptic potentiation [10], [11], [12], cortical responses after crossing nerve transfer were analyzed in cortex-restricted heterozygous GluN1 (NMDAR1) knockout mice [13]. In mice that express both Cre and LoxP, approximately 50% of functional NMDA receptors are specifically lost in cortical excitatory neurons [13], while no apparent abnormality has been found during development. In control mice that expressed Cre only, LoxP only, or neither, bilateral cortical representation was observed at 8 weeks after crossing nerve transfer (Fig. 6Aa). However, in mice that expressed both Cre and LoxP, bilateral cortical representation was less obvious (Fig. 6Ab). Response amplitudes were measured in the ipsilateral right S1 and contralateral right S1 (Fig. 6B, C). Ipsilateral S1 responses were slightly, but significantly reduced in mice that expressed both Cre and LoxP compared with those in the control mice (P<0.05). Contralateral S1 responses were much more significantly reduced (P<0.002). As a result, bilaterality index in cortex-restricted heterozygous GluN1 knockout mice was significantly reduced by approximately 50% compared to the control mice (P<0.02), but remained significantly larger than the bilaterality index in naïve mice (P<0.02, Fig. 6D). These results suggest that the extent of bilateral cortical representation in cortex-restricted heterozygous GluN1 knockout mice was reduced to approximately 50% of the control value.

### Crossing nerve transfer in protocadherin-α knockout mice

**Figure 7:**
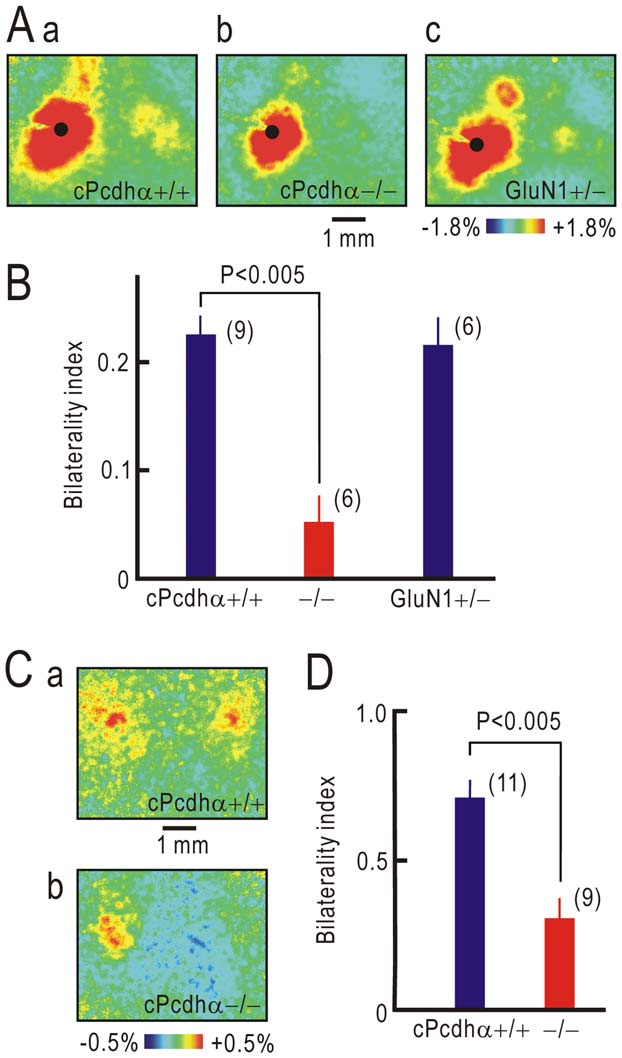
Crossing nerve transfer in cPcdhα knockout mice. (A) Cortical responses elicited by transcranial stimulation of the left S1 in control cPcdhα +/+ (a) and cPcdhα −/− mice (b). Responses in a cortex-restricted GluN1 +/− mouse are also shown for comparison (c). (B) Bilaterality index of cortical responses elicited by transcranial stimulation of the left S1 in cPcdhα +/+, cPcdhα −/−, and cortex-restricted GluN1 +/− mice. (C) Cortical responses elicited by left forepaw stimulation at 8 weeks after crossing nerve transfer in cPcdhα +/+ (a) and cPcdhα −/− mice (b). (D) Bilaterality index after crossing nerve transfer in cPcdhα +/+ and cPcdhα −/− mice.

Protocadherins are neuron-specific cell adhesion molecules that are thought be involved in synaptic formation [22], [23]. We utilized mice with a mutation in the constant region of protocadherin-α (cPcdhα), because these mice exhibit abnormalities in some synaptic pathways [24], [25]. Cortico-cortical pathways between S1 in both hemispheres were impaired in cPcdhα knockout mice, and the bilaterality index obtained from cortical responses elicited by transcranial cortical stimulation [26] was significantly less in these mice compared with control mice with normal cPcdhα (P<0.005, Fig. 7A, B). As expected, cortical responses in the contralateral S1 were not clearly observed at 8 weeks after crossing nerve transfer in Pcdhα knockout mice (Fig. 7C), and the bilaterality index was significantly less than in control mice with normal cPcdhα (Fig. 7D). These results confirmed the importance of cortico-cortical pathways between S1 in both hemispheres for S1 bilateral cortical representation after crossing nerve transfer. Interestingly, the bilaterality index obtained from cortical responses elicited by transcranial cortical stimulation in cortex-restricted heterozygous GluN1 knockout mice was comparable to normal mice (Fig. 7B), suggesting that the mechanisms underlying reduced bilateral cortical representation after crossing nerve transfer were different between cPcdhα knockout mice and cortex-restricted heterozygous GluN1 knockout mice.

## Discussion

### Bilateral cortical representation in S1 after crossing nerve transfer

Results from the present study demonstrated that cortical activity elicited by forepaw stimulation in mice was restored in the contralateral S1 after crossing nerve transfer. However, peripheral nerve injury is sufficient to induce reorganization of somatosensory cortical maps within a hemisphere [7], [8], [27]. Resection of the median and ulnar nerves, which innervate the forepaw palm, has been shown to induce sensitization of the remaining radial and musculocutaneous nerves within a few hours, and contralateral cortical responses to the applied stimulation apparently recover [19]. To exclude the contribution of denervation-induced sensitization of the remaining radial and musculocutaneous nerves, the median and ulnar nerves were cut as well as the radial and musculocutaneous nerves, prior to imaging experiments. In addition, the present results confirmed that crossing nerve transfer of the median and ulnar nerves, but not denervation-induced sensitization of remaining nerves, was responsible for bilateral cortical representation, because the bilateral cortical responses were abolished by cutting the nerve graft. In a previous study, electrophysiological recordings did not detect contralateral S1 activity elicited by electrical stimulation of peripheral nerves [28]. However, contralateral cortical responses were recorded in the present study, probably because the present imaging technique was suitable for detecting polysynaptic, unsynchronized cortical activity elicited by vibratory stimuli and distributed over a wide cortical area. Bilateral cortical representation in S1 was established at 8 weeks after crossing nerve transfer, and no further change was found in S1 up to 12 months after the operation. These time-course changes were much quicker than recovery of motor functions after crossing nerve transfer [5], [6], probably because functional modification of cortico-cortical pathways between S1 in both hemispheres was sufficient for S1 recovery but not recovery of motor functions [5]. It is possible that motor recovery correlated with preceding changes in S1. Anyway, these results suggested that restoration of cortical activity in the contralateral S1 in mice is compatible with sensory recovery in patients treated with crossing nerve transfer.

### Activity-dependent potentiation of cortical synapses

The appearance of cortical responses in the ipsilateral S1 after crossing nerve transfer was easily expected, because primary sensory neurons projecting to the ipsilateral S1 have an inherent capacity to regenerate of their lesioned axons, and the regenerated axons are led to the lesioned forepaw via the nerve graft used for crossing nerve transfer [29]. The ipsilateral S1 responses observed at 4 weeks after crossing nerve transfer confirmed this expectation. However, restoration of cortical activity in the contralateral S1 at 8 weeks after crossing nerve transfer required further analysis. Cortical stimulation applied to S1 produces neural activities in the contralateral S1 via cortico-cortical pathways [9]. Involvement of cortico-cortical pathways in bilateral cortical representation was suggested by results from the photo-inactivation experiment, which demonstrated that cortical activity in the contralateral S1 was secondarily produced by ipsilateral S1 activity. In cPcdhα knockout mice with impaired connections between S1 in both hemispheres, weak contralateral cortical activity after crossing nerve transfer provided further support for the role of cortico-cortical pathways. Cortical synapses in S1 could be potentiated by crossing nerve transfer depending on NMDA receptors [30], because the bilaterality index after crossing nerve transfer was reduced in cortex-restricted heterozygous GluN1 knockout mice. Previous results have suggested dose-dependent effects of NMDA receptors [11]; therefore, bilateral cortical representation should be almost completely abolished if all GluN1 subunits were removed from the cortex. The bilaterality index obtained from cortical responses elicited by transcranial stimulation in cortex-restricted heterozygous GluN1 knockout mice was comparable to normal mice, suggesting that functional modification after crossing nerve transfer, rather than formation of cortico-cortical synapses prior to surgery was dependent on NMDA receptors.

**Figure 8:**
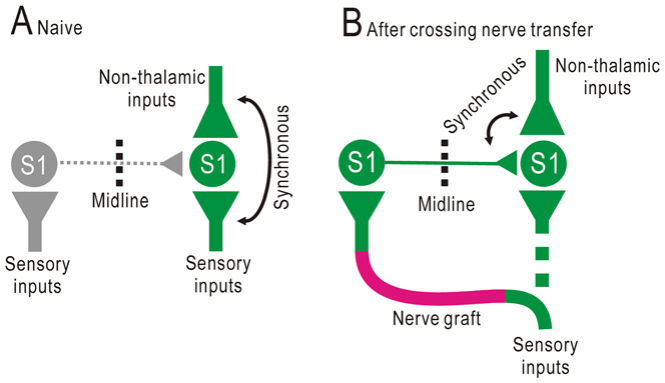
Activity-dependent mechanisms underlying bilateral cortical representation. (A) Neural circuits prior to crossing nerve transfer. Cortico-cortical inputs from the left S1 to the right S1 are not necessarily synchronous to other non-thalamic inputs to the right S1, because sensory inputs from both forepaws are not necessarily synchronous. In contrast, thalamic inputs to the right S1 are synchronous to other non-thalamic inputs to the right S1. (B) Neural circuits after crossing nerve transfer. Cortico-cortical inputs from the left S1 to the right S1 are now synchronous to other non-thalamic inputs to the right S1, so that the cortico-cortical inputs can be synchronized with postsynaptic activities in the right S1. Cortico-cortical synapses from the left S1 to the right S1 are potentiated according to the Hebbian rule.

NMDA receptors play an essential role in induction of synaptic potentiation that is dominated by the Hebbian rule [10]. Therefore, potentiation of cortico-cortical synapses after crossing nerve transfer could also be induced according to the Hebbian rule. Timing of synaptic inputs is crucial for somatosensory plasticity [31]. Cortical activity in S1 is produced by somatosensory thalamic inputs and other non-thalamic inputs, and synchronization between these inputs explains the modification of postsynaptic activity in S1 according to attention or behavioral states [32], [33]. In naïve mice, sensory inputs to each side of S1 are independent from each other, and the cortico-cortical inputs to S1 cannot be synchronized to other non-thalamic inputs (Fig. 8A). After crossing nerve transfer, however, cortico-cortical inputs from the ipsilateral S1 can be synchronized to non-thalamic inputs of the contralateral S1 depending on the attention and/or behavioral state, and may be potentiated according to the Hebbian rule (Fig. 8B). This hypothesis was supported by our finding that bilateral cortical representation was induced by direct stimulation applied to the ipsilateral S1 and paired with forepaw stimulation. Induced potentiation by cortical stimulation was typically not maintained for more than 40 min, which was consistent with previous results [9]. However, bilateral representation that lasted for more than 80 min was observed in some mice, suggesting that transient potentiation may be converted to a more stable form through repeated pairing between cortico-cortical inputs and other non-thalamic inputs in behaving mice with crossing nerve transfer. Taken together, the present results suggest that restoration of the contralateral cortical activity in S1 after crossing nerve transfer was attributed to activity-dependent potentiation of cortico-cortical synapses between S1 in both hemispheres.

### Functional sensory recovery after crossing nerve transfer

When BP injuries are repaired by nerve transfer between the injured nerve and accessory nerve or intercostal nerve [2], new pathways must be produced for functional recovery with potential rewiring errors [1]. The rewiring error could be avoided in crossing nerve transfer, because neurite extensions of primary sensory neurons and functional modification of cortico-cortical pathways are sufficient for restoration of S1 contralateral activity. Cortico-cortical pathways that connect both sides of S1 play important roles in sensory information transfer between both sides of S1 corresponding to body parts where bilateral coordination is essential [34], [35], [36], was well as in midline areas, such as the intraoral cavity, chin, or trunk [37]. Although it is difficult to reverse left-right side of somatosensory inputs to the brain without crossing nerve transfer, retinal image reversal is easily achieved through the use of prism spectacles [38]. Reversed visual inputs induce adaptation to reversed vision together with bilateral cortical representation in the visual cortex [39], [40]. Therefore, crossing nerve transfer might also result in functional adaptation to reversed somatosensory inputs. However, a significant part of the ipsilateral S1 in mice was stimulated by forepaw stimulation with crossing nerve transfer in an end-to-end fashion, and synchronized sensation from injured and healthy limbs has been previously reported in patients after crossing nerve transfer in an end-to-end fashion [28].

Restoration of contralateral cortical activity was more efficient in mice operated in an end-to-side fashion. Our research group examined a patient case operated in an end-to-side fashion. The patient exhibited avulsion injury to the right C7, C8, and Th1 roots, as well as laceration of the right upper trunk of BP (C5 and C6 roots), as a result of a motorcycle accident. Surgery was performed in an end-to-side fashion at 43 days after the accident. At six months after surgery, tapping of the nerve graft in the cervical subcutaneous tunnel resulted in a radiating pain sensation in the right thumb, with no tingling sensation on the contralateral left side. At nine years after surgery, tapping of the graft resulted in pain sensation in the right thumb, index finger, and palm with no tingling sensation in the contralateral left side. Subcutaneous injection of mepivacane, a local anesthetic, at the nerve graft locating in the anterior neck resulted in a transient sensory loss in the right forearm and hand, with no apparent effect on the contralateral left side. However, it is unknown why the unreversed sensation, rather than a synchronized sensation, was achieved in this patient by crossing nerve transfer in an end-to-side fashion. Somatosensory information processing in S1 is followed by processing in the secondary somatosensory cortex [41], and S1 changes after crossing nerve transfer might be followed by changes in the secondary area [42]. Therefore, functional sensory recovery after crossing nerve transfer could be affected by neural plasticity not only in S1 but also in these higher areas. The unreversed sensation in the patient might be explained, if cortical activity in the higher areas is recovered only in the contralateral hemisphere after crossing nerve transfer in an end-to-side fashion. This expectation remains to be tested in future studies.

## Materials and Methods

The present study was performed according to the guidelines for animal experiments of Niigata University and had the approval of the ethics committee of Niigata University. Male C57BL/6 mice were used. Cortex-restricted heterozygous GluN1 knockout mice [13] and cPcdhα knockout mice [24], which were based on the C57BL/6 strain, were also used.

### Crossing nerve transfer

Mice were anesthetized at 8 weeks of age with pentobarbital (40 mg/kg, i.p.), and fastened in a supine position on a flat surface. BPs of both sides were exposed after an infraclavicular hockey stick incision of the skin under sterile conditions. The pectoralis major muscle was split along the arcuate line, and the pectoralis minor muscle was laterally retracted. The medial cord, median nerve, and ulnar nerve were identified under a binocular microscope. On the recipient side (left), the medial cord was cut at the level just proximal to the point where the medial cord diverges into the median nerve and ulnar nerve. When nerve transfer surgery was performed in an end-to-end fashion (Fig. 2), the medial cord of the donor side (right) was cut at the level distal to the pectoralis branch origin. A sciatic nerve graft (2 cm long) was harvested from another mouse anesthetized with pentobarbital. The cut ends of the nerves were connected to the nerve graft with 11-0 sutures. The left radial and musculocutaneous nerves were also cut at the same time, because left forepaw stimulation has been shown to produce cortical responses in the contralateral S1 via these nerves [19]. In the second group of mice, the left radial and musculocutaneous nerves were cut at 7 weeks after initial surgery under pentobarbital anesthesia (sequential nerve cut). In the third group of mice, the nerve graft and an epineural sheath window on the right BP were sutured in an end-to-side fashion to reduce surgical damage to the donor side [20]. In this group of mice, nerve transfer and left radial and musculocutaneous nerve cutting were performed at the same time. The nerve graft and BPs were covered by suturing the skin. Fradiomycin (Mochida Pharmaceutical, Tokyo, Japan) and ampicillin (Meiji Seika, Tokyo, Japan) were used to avoid infection.

### Flavoprotein fluorescence imaging

Imaging experiments were performed as previously described [19]. Mice were anesthetized with urethane (1.7 g/kg, i.p.) at 8 weeks after crossing nerve transfer, unless otherwise specified. Rectal temperature was monitored and maintained at 38°C using a heating pad throughout the experiments. After the disinfected skin was removed, the skull at S1 was exposed and covered with 2% agarose (Type I-B; Sigma-Aldrich, St. Louis, U.S.A.) dissolved in saline. Cortical images (128×160 pixels) of green (λ = 500–550 nm) fluorescence in blue (λ = 450–490 nm) excitation light were recorded using a cooled CCD camera system (AQUACOSMOS/Ratio system with a ORCA-ER camera; Hamamatsu Photonics, Hamamatsu, Japan). The camera was attached to a binocular epifluorescence microscope (MZ FL III; Leica Microsystems, Wetzlar, Germany) with an objective lens (magnification: 1.0, numerical aperture: 0.125). To elicit fluorescence responses, sinusoidal vibration (displacement: ±0.4 mm, 50 Hz for 0.5 s) was produced with a mechanical stimulator (DPS-270; Dia Medical, Tokyo, Japan), and applied with a brush to the left plantar forepaw surface. Fluorescence images were obtained at 9 frames/s, which were averaged over 24 or 40 trials. Moving spatial averaging in 5 by 5 pixels, and temporal averaging in 3 consecutive frames, were used for smoothing and improving image quality. The normalized images were shown in a pseudocolor scale in terms of relative fluorescence changes (ΔF/F_0_), which were obtained by dividing increased in fluorescence intensity (ΔF) in each pixel by averaged intensity in 5 frames immediately prior to stimulation (F_0_). Response amplitude was evaluated as ΔF/F_0_ in a square window of 10 by 10 pixels (0.36 by 0.36 mm) including the response peak, which was found by visual inspection in serial pseudocolor images. The window location was adjusted, so that the response amplitude in the window was maximal. When ΔF/F_0_ was measured in a hemisphere with almost no response, the window was placed at the location symmetrical to the response area in the contralateral hemisphere. To estimate the extent of bilateral cortical representation in S1, bilaterality index was calculated as the ratio of response amplitudes in both hemispheres. In naïve mice, it was defined as ipsilateral response amplitudes normalized by contralateral amplitudes. In mice with crossing nerve transfer, the bilaterality index was defined as contralateral response amplitudes normalized by ipsilateral amplitudes. After the imaging experiments, the mice were sacrificed with an overdose of pentobarbital (i.p.). Using a binocular microscope, it was confirmed that the left forepaw of each mouse with crossing nerve transfer was connected to the brain via the nerve graft only.

### Photo-inactivation of cortical responses

Cortical activities in S1 were transcranially suppressed by photobleaching of flavoproteins and resulting suppression of aerobic energy metabolism, as previously described [21]. A solid state blue (λ = 475 nm) laser of 20 mW (BWB475-20; B&W Tek, Newark, U.S.A.) was used to induce photo-inactivation of neural activity in anesthetized mice. The laser beam size (1/e^2^) was approximately 1 mm or less. The tip of the light guide attached to the laser was placed approximately 1 cm away from the skull surface, so that an area with a diameter of approximately 1 mm was illuminated. Irradiation was maintained for 90 min, and neural activity was compared before and immediately after light exposure.

### Direct cortical stimulation

To produce S1 bilateral cortical representation in naïve mice, direct cortical stimulation at 100 Hz was applied for 1 s to the left S1 around the forepaw area [9]. Current pulses, with a duration of 100 µs and intensity of 300 µA, were applied via an electrolytically polished tungsten electrode, which was insulated with polyvinyl chloride (except for the region within 60–90 µm of the tip). The stimulus electrode was inserted though a hole in the skull to a depth of 800 µm from the pial surface. To facilitate cortical stimulation effects, 50 Hz vibratory stimulation was applied to the left forepaw for 1 s in conjunction with cortical stimulation. After cortical stimulation was repeated 10 times at 1 min intervals, cortical responses to forepaw stimulation alone were recorded within 20 min after cessation of cortical stimulation. This combination was repeated several times. Finally, cortical responses to forepaw stimulation alone were recorded at more than 40 min after cessation of cortical stimulation.

When functional cortico-cortical connection between S1 in both hemispheres was estimated, the forepaw area was transcranially stimulated, as previously described [26]. Briefly, the skull was shaved with a blade on a dental drill so that the thinned skull was easily deformed when slight force was applied. The blunt tip of a sewing needle, with a diameter of approximately 100 µm, was pressed on to the shaved skull so that the subarachnoid space around the tip was compressed. Current pulses at 10 Hz, with an intensity of 500 µA, were applied for 1 s to the needle to elicit localized cortical activity in the forepaw area of S1. To estimate the extent of functional connections between both hemispheres, bilaterality index was calculated as amplitudes of contralateral S1 responses in the normalized by response amplitudes around the stimulated sites.

### Statistical analysis

Statistical significance was tested using StatView software (SAS Institute, Cary, USA). Differences between unpaired data were evaluated using the Mann-Whitney U test, and differences in paired data were evaluated using the Wilcoxon signed rank test. Only significant differences (P<0.05) are shown in the figures.

## References

[1] Lundborg G, Rosén B (2007). Hand function after nerve repair.. Acta Physiol (Oxf).

[2] Addas BM, Midha R (2009). Nerve transfers for severe nerve injury.. Neurosurg Clin N Am.

[3] Gu YD, Zhang GM, Chen DS, Yan JG, Cheng XM (1992). Seventh cervical nerve root transfer from the contralateral healthy side for treatment of brachial plexus root avulsion.. J Hand Surg.

[4] Gu YD, Shen LY (1994). Electrophysiological changes after severance of the C7 nerve root.. J Hand Surg.

[5] Lou L, Shou T, Li Z, Li W, Gu Y (2006). Transhemispheric functional reorganization of the motor cortex induced by the peripheral contralateral nerve transfer to the injured arm.. Neuroscience.

[6] Zuo CT, Hua XY, Guan YH, Xu WD, Xu JG (2010). Long-range plasticity between intact hemispheres after contralateral cervical nerve transfer in humans.. J Neurosurg.

[7] Merzenich MM, Kaas JH, Wall J, Nelson RJ, Sur M (1983). Topographic reorganization of somatosensory cortical areas 3b and 1 in adult monkeys following restricted deafferentation.. Neuroscience.

[8] Jain N, Qi HX, Collins CE, Kaas JH (2008). Large-scale reorganization in the somatosensory cortex and thalamus after sensory loss in macaque monkeys.. J Neurosci.

[9] Murakami H, Kamatani D, Hishida R, Takao T, Kudoh M (2004). Short-term plasticity visualized with flavoprotein autofluorescence in the somatosensory cortex of anesthetized rats.. Eur J Neurosci.

[10] Bliss TVP, Collingridge GL (1993). A synaptic model of memory: long-term potentiation in the hippocampus.. Nature.

[11] Tang YP, Shimizu E, Dube GR, Rampon C, Kerchner GA (1999). Genetic enhancement of learning and memory in mice.. Nature.

[12] Citri A, Malenka RC (2008). Synaptic plasticity: multiple forms, functions, and mechanisms.. Neuropsychopharmacology.

[13] Iwasato T, Datwani A, Wolf AM, Nishiyama H, Taguchi Y (2000). Cortex-restricted disruption of NMDAR1 impairs neuronal patterns in the barrel cortex.. Nature.

[14] Schuett S, Bonhoeffer T, Hubener M (2002). Mapping retinotopic structure in mouse visual cortex with optical imaging.. J Neurosci.

[15] Chance B, Cohen P, Jöbsis FF, Schoener B (1962). Intracellular oxidation-reduction states in vivo.. Science.

[16] Shibuki K, Hishida R, Murakami H, Kudoh M, Kawaguchi T (2003). Dynamic imaging of somatosensory cortical activities in the rat visualized by flavoprotein autofluorescence.. J Physiol (Lond).

[17] Takahashi K, Hishida R, Kubota Y, Kudoh M, Takahashi S (2006). Transcranial fluorescence imaging of auditory cortical plasticity regulated by acoustic environments in mice.. Eur J Neurosci.

[18] Tohmi M, Kitaura H, Komagata S, Kudoh M, Shibuki K (2006). Enduring critical period plasticity visualized by transcranial flavoprotein imaging in mouse primary visual cortex.. J Neurosci.

[19] Komagata S, Chen S, Suzuki A, Yamashita H, Hishida R (2011). Initial phase of neuropathic pain within a few hours after nerve injury in mice.. J Neurosci.

[20] Viterbo F, Trindade JC, Hoshino K, Mazzoni A (1994). Two end-to-side neurorrhaphies and nerve graft with removal of the epineural sheath: experimental study in rats.. Br J Plast Surg.

[21] Kubota Y, Kamatani D, Tsukano H, Ohshima S, Takahashi K (2008). Transcranial photo-inactivation of neural activities in the mouse auditory cortex.. Neurosci Res.

[22] Kohmura N, Senzaki K, Hamada S, Kai N, Yasuda R (1998). Diversity revealed by a novel family of cadherins expressed in neurons at a synaptic complex.. Neuron.

[23] Schreiner D, Weiner JA (2010). Combinatorial homophilic interaction between gamma-protocadherin multimers greatly expands the molecular diversity of cell adhesion.. Proc Natl Acad Sci USA.

[24] Hasegawa S, Hamada S, Kumode Y, Esumi S, Katori S (2008). The protocadherin-alpha family is involved in axonal coalescence oolfactory sensory neurons into glomeruli of the olfactory bulb in mouse.. Mol Cell Neurosci.

[25] Katori S, Hamada S, Noguchi Y, Fukuda E, Yamamoto T (2009). Protocadherin-α family is required for serotonergic projections to appropriately innervate target brain areas.. J Neurosci.

[26] Hishida R, Watanabe K, Kudoh M, Shibuki K (2011). Transcranial electrical stimulation of cortico-cortical connections in anesthetized mice.. J Neurosci Meth.

[27] Kitaura H, Hishida R, Shibuki K (2010). Transcranial imaging of somatotopic map plasticity after tail cut in mice.. Brain Res.

[28] Wang M, Li ZY, Xu WD, Hua XY, Xu JG (2010). Sensory restoration in cortical level after a contralateral C7 nerve transfer to an injured arm in rats.. Neurosurgery.

[29] Donnerer J (2003). Regeneration of primary sensory neurons.. Pharmacology.

[30] Watanabe K, Kamatani D, Hishida R, Kudoh M, Shibuki K (2007). Long-term depression induced by local tetanic stimulation in the rat auditory cortex.. Brain Res.

[31] Wang X, Merzenich MM, Sameshima K, Jenkins WM (1995). Remodelling of hand representation in adult cortex determined by timing of tactile stimulation.. Nature.

[32] Ferezou I, Bolea S, Petersen CC (2006). Visualizing the cortical representation of whisker touch: voltage-sensitive dye imaging in freely moving mice.. Neuron.

[33] Wiest MC, Thomson E, Pantoja J, Nicolelis MA (2010). Changes in S1 neural responses during tactile discrimination learning.. J Neurophysiol.

[34] Iwamura Y, Iriki A, Tanaka M (1994). Bilateral hand representation in the postcentral somatosensory cortex.. Nature.

[35] Iwamura Y, Tanaka M, Iriki A, Taoka M, Toda T (2002). Processing of tactile and kinesthetic signals from bilateral sides of the body in the postcentral gyrus of awake monkeys.. Behav Brain Res.

[36] Fabri M, Del Pesce M, Paggi A, Polonara G, Bartolini M (2005). Contribution of posterior corpus callosum to the interhemispheric transfer of tactile information.. Brain Res Cogn Brain Res.

[37] Fabri M, Polonara G, Mascioli G, Paggi A, Salvolini U (2006). Contribution of the corpus callosum to bilateral representation of the trunk midline in the human brain: an fMRI study of callosotomized patients.. Eur J Neurosci.

[38] Shimojo S, Nakajima Y (1981). Adaptation to the reversal of binocular depth cues: effects of wearing left-right reversing spectacles on stereoscopic depth perception.. Perception.

[39] Sugita Y (1996). Global plasticity in adult visual cortex following reversal of visual input.. Nature.

[40] Tanaka Y, Miyauchi S, Misaki M, Tashiro T (2007). Mirror symmetrical transfer of perceptual learning by prism adaptation.. Vision Res.

[41] Romo R, Salinas E (2001). Touch and go: decision-making mechanisms in somatosensation.. Annu Rev Neurosci.

[42] Kamatani D, Hishida R, Kudoh M, Shibuki K (2007). Experience-dependent formation of activity propagation patterns at the somatosensory S1 and S2 boundary in rat cortical slices.. Neuroimage.

